# The Treatment of Chronic Dyspepsia with H_2_O_2_

**Published:** 1900-04

**Authors:** 


### The Treatment of Chronic Dyspepsia with $H_2O_2$.

Gilbert (*N. E. Med. Monthly*) reports the following case:

Peter H., aged 40, Hungarian, farm laborer, applied for treatment at my office on July 1, 1899. He was a strapping fellow, mostly skin and bones, of about 170 pounds weight, and would not have been thought ill except for the prominent dark rings under his eyes, his injected conjunctivæ, and a drawn, haunted expression on his countenance, indicative of past trouble or imminent danger. The history he gave was somewhat as follows:

Six years previously, on his voyage to this country, he suffered from an attack of acute gastritis, attended with retchings of the most violent character. Soon after landing he recovered sufficiently to attend to his work; but he says he has never been the same man since. In all this long period he has not eaten a good meal, nor enjoyed what he has eaten, the burning pain in the epigastrium, after meals, becoming so great occasionally that for fear of its repetition he has gone without food for two or three days at a time. Belching of enormous quantities of gas, too, is common with him soon after eating, thus evidencing the presence of undigested food with its resultant fermentation. The patient states, that in order to get relief he has spent all of his wages upon various doctors, specialists, quacks, nostrums, etc., and declares that he is worse today than on the day he first landed in this country.

On examination it was found that he was slightly feverish, pulse rapid, tongue flabby and heavily coated, while the teeth and entire cavity of the mouth were covered with a foul-smelling sticky mucus. That the stomach received, in the process of starch digestion, little or no assistance from the salivary glands of the mouth was plainly apparent. In deciding on the mode of treatment it was obvious that lack of the usual amount of gastric secretion must be met by restoring the physiological conditions upon which the secretion depends. In other words, in order to relieve the inflammatory condition of the gastric mucous membrane and restore the function of the peptic glands, antiseptics were required. The patient therefore was furnished with a flask of ozonized water, made of one part hydrozone to four parts of water, and directed to wash out his mouth every night and morning, thoroughly cleansing the tongue, teeth and gums of the unhealthy mucus and any pathogenic germs it might contain. To destroy the microbic elements of fermentation in the stomach and dissolve the tenacious mucus there, a mixture of one ounce hydrozone with two quarts of sterilised water was made, and half a tumblerful directed to be taken half an hour before meals. Having thus procured a clean surface in the stomach, the patient was advised to take immediately after meals, a drachm of glycozone, diluted in a wine-glassful of water, for the purpose of enhancing cellular action and stimulating healthy granulations. Of course, he was ordered to select his food with care and eat regularly.

The result of this simple procedure was magical. Although for the first two or three days there was some discomfort after eating, this soon disappeared, and at the end of a fortnight the patient reported that for the first time in six years he was enabled to eat his meals without dread of subsequent distress and eructations of gas. (In the opinion of the writer the fermentation was thus quickly subdued by the active oxidation resulting from the liberation of nascent oxygen.) The treatment was continued in this manner for another month and then gradually abandoned. On September 1st, the patient reported, expressed his gratefulness, said that he weighed 185 pounds and believed himself to be completely cured.

